# Review: Challenges of In Vitro CAF Modelling in Liver Cancers

**DOI:** 10.3390/cancers13235914

**Published:** 2021-11-24

**Authors:** Alba Herrero, Elisabeth Knetemann, Inge Mannaerts

**Affiliations:** 1 Department of Cell Biology and Histology, School of Medicine and Nursing, University of the Basque Country (UPV/EHU), 48940 Leioa, Bizkaia, Spain; alba.herrero.alonso@vub.be; 2 Liver Cell Biology Research Group, Faculty of Medicine and Farmacy, Vrije Universiteit Brussels, 1090 Brussels, Belgium; elisabeth.knetemann@vub.be

**Keywords:** liver cancer, hepatic stellate cells, cancer-associated fibroblasts, in vitro models, 2D, 3D

## Abstract

**Simple Summary:**

Liver cancer and tumours spreading from other organs to the liver are associated with high death rates. Current treatments include surgical removal of the tumour and chemotherapy. Unfortunately, patients are often re-diagnosed with liver nodules in the years after cessation of the treatment. Therefore, scientists are looking for alternative treatment strategies, and these include targeting the tumour environment. The tumour environment includes the cancer-associated fibroblasts, which could be an interesting target for therapy in combination with current strategies. In this review paper we summarize the current models to investigate the effect of the tumour on the cancer-associated fibroblasts. Not many studies focus on the cancer-associated fibroblasts in non-animal models and this should improve in order to better understand the role of the cancer-associated fibroblasts and to evaluate the potential of cancer-associated fibroblast-directed therapies.

**Abstract:**

Primary and secondary liver cancer are the third cause of death in the world, and as the incidence is increasing, liver cancer represents a global health burden. Current treatment strategies are insufficient to permanently cure patients from this devastating disease, and therefore other approaches are under investigation. The importance of cancer-associated fibroblasts (CAFs) in the tumour microenvironment is evident, and many pre-clinical studies have shown increased tumour aggressiveness in the presence of CAFs. However, it remains unclear how hepatic stellate cells are triggered by the tumour to become CAFs and how the recently described CAF subtypes originate and orchestrate pro-tumoural effects. Specialized in vitro systems will be needed to address these questions. In this review, we present the currently used in vitro models to study CAFs in primary and secondary liver cancer and highlight the trend from using oversimplified 2D culture systems to more complex 3D models. Relatively few studies report on the impact of cancer (sub)types on CAFs and the tumour microenvironment, and most studies investigated the impact of secreted factors due to the nature of the models.

## 1. Introduction

The liver is an essential organ for human physiology and is composed of two major groups of cells: parenchymal cells, or hepatocytes, and non-parenchymal cells, such as liver sinusoidal endothelial cells, hepatic stellate cells (HSCs), and Kupffer cells [1]. These cell types are critical for the liver to maintain the physiological homeostasis of the human body by detoxifying blood, metabolising macronutrients, maintaining lipid and cholesterol levels, and providing support to the immune system [2]. In order to carry out these functions, the liver is always in an immune-depressed state, which makes it susceptible to the development of primary liver cancer or to harbouring metastases from other organs such as colon [3], breast [4], lung [5], or skin [6].

Curative therapy for liver cancer (i.e., surgical resection, liver transplantation, and/or local ablation) is most effective at the early stages of the disease, so the currently available treatment strategies remain unsatisfactory for advanced disease. Tumour resection is only possible for a small proportion of metastatic patients and around 75% of patients undergo rapid relapse [7]. A better understanding of the underlying molecular mechanisms that drive tumour progression and metastasis could provide us with new treatment options. As the malignant behaviour of tumour cells can be greatly modulated by the tumour microenvironment (TME), knowledge about and targeting of the TME offers new therapeutic opportunities [7,8,9,10,11,12].

## 2. Epidemiology of Liver Cancer

### 2.1. Primary Liver Cancer

Primary liver cancer is the sixth most frequently diagnosed cancer and the third leading cause of cancer-related death worldwide (see International Agency of Research Cancer statistics). Within primary liver cancer, hepatocellular carcinoma (HCC) is the most common type, representing 75–85% of all primary liver cancer cases (see International Agency of Research Cancer statistics). Asia is the leading continent concerning HCC incidence, prevalence, and mortality followed by Europe [13]. Risk factors for HCC include hepatitis B virus, hepatitis C virus, consumption of aflatoxin B1 (AFB1)-contaminated food, excessive consumption of alcohol, and non-alcoholic fatty liver disease [14]. All these risk factors induce liver fibrosis, characterized by a progressive accumulation of extracellular matrix (ECM), which is the main determinant for liver cancer prognosis [15]. Continued exposure to these risk factors leads to the development of liver cirrhosis, which is strongly related to HCC progression [16].

Liver cancer cases caused by hepatitis B and C viruses in countries with high sociodemographic indexes are 18% and 40% of absolute liver cancer deaths, respectively [17]. On the other hand, high exposure to AFB1, a chemically stable mycotoxin with a strong carcinogenic potential present in several types of food [18], is estimated to cause around 5–28% of global HCC cases [19]. Lastly, alcoholic and non-alcoholic fatty liver disease are the most prevalent types of liver disease worldwide and are strongly related to the development of HCC and other extrahepatic cancers such as colorectal cancer or breast cancer [20].

The current treatment options for HCC are partial hepatectomy, liver transplantation, or chemotherapy [21]. However, the 5-year survival rate for liver cancer is only 18%, a very low percentage that shows the necessity for new approaches to battle this disease [22].

### 2.2. Secondary Liver Cancer

Besides being highly susceptible to the development of primary cancer, the liver is also the main organ to suffer metastases from several cancer types such as colorectal cancer (CRC) [3], breast cancer [4], lung cancer [5], or skin cancer [6]. In CRC liver metastases (CRLM), tumour cells have the capacity to intravasate into the blood stream [23] and invade the healthy liver [24].

CRC is the third leading cancer type in incidence and the second leading cancer type in mortality and prevalence worldwide [25,26]. CRC can be divided in four subtypes based on clinical level, origin, and pathophysiology [27,28,29,30]. The most common subtype is adenocarcinoma, which originates from the epithelial cells that reside in the colon [28]. This subtype has a low survival rate and only approximately 25% of cases are operable [28]. Among patients with CRC, 15–25% suffer from CRLM at the time of diagnosis, and approximately 50% develop them at some point during the disease progression [31]. Moreover, 70% of the CRC-related deaths are caused by CRLM [32]. Although treatment has improved substantially in recent decades, to date, surgical resection of the liver metastases is the only treatment option that significantly increases the survival time [33]. However, only a minority of patients are suitable for surgery [34], and of all patients undergoing surgery, 30% present with new metastases and in 15% of the cases the patient dies less than a year after the operation [33].

## 3. Role of Microenvironment in Liver Tumour Development

**Figure 1:**
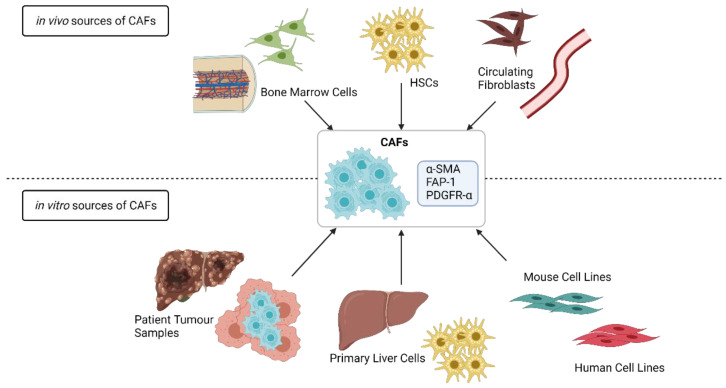
Cellular sources of myofibroblasts or CAFs in liver cancer. CAFs are a heterogeneous group of cells derived from several sources. In the liver, the majority of CAFs are derived from HSCs, but a minor percentage of CAFs originates from Bone Marrow Cells or Circulating Fibroblasts. The most frequently used markers to define CAFs include α-SMA, FAP-1, and PDGFR-α and are shared by the different cellular CAF sources. For in vitro studies, fibroblast or HSC cell lines of mouse or human origin are often used, but some studies use CAFs isolated from Mouse or Patient Tumour Samples or Primary Hepatic Stellate Cells. Image created by Biorender.

Solid tumours consist of cellular and acellular components which form the TME, as reviewed by Baghban et al. and Arneth et al. [35,36]. Primary and secondary liver tumours display different timings of TME appearance. In HCC, the TME is formed at the same time as the tumour, supporting the growth of the tumour cells [37]. In secondary liver cancer, several studies have shown that the tumour cells prepare the liver for the arrival of metastases creating a pre-metastatic niche through the release of tumour exosomes or small vesicles [38,39]. While the TME formation differs between primary and secondary liver cancer, the overall composition is the same. The liver TME is composed of liver resident cells such as liver sinusoidal endothelial cells, HSCs, and Kupffer cells [35] and of other cell types such as myeloid-derived suppressor cells, regulatory T-cells, tumour-associated macrophages, and cancer-associated fibroblasts (CAFs) [39,40]. Recent studies have shown that HSCs are the major source of CAFs in liver cancer, although a small portion of the CAFs can be derived from other cell types, as shown in Figure 1 [9,41].

Recently, it was demonstrated that HSC-derived CAFs are essential for the production and remodelling of ECM in liver tumours [9,42]. An injured liver is characterized by the accumulation of ECM, which is composed of type I and III fibrillar collagens, fibronectin, undulin, laminin, hyaluronan, elastin, and proteoglycans [43]. Furthermore, Jia Y.L et al. showed that the expression of epimorphin by CAFs promotes the expression of MMP9 in the liver TME, which increases the invasive capacity of the cancer cells [44]. In addition, Jia C et al. uncovered that IL-6 mediates the promotion of endothelial–mesenchymal transition (EMT) in HCC cells by CAFs through the activation of the IL-6/IL6-R/STAT3 signalling pathway, which in turn promotes the expression of transglutaminase 2 [45], a protein which is strongly related to EMT and upregulated in HCC [46].

Besides the modulation of the ECM, CAFs are also involved in tumour growth [47], survival [48], and chemoresistance [49]. For example, Song et al. found that CAFs suppress cancer cell apoptosis through the activation of the SDF-1/CXCR4/PI3K/AKT pathway, which leads to an increased Bcl2/BAX ratio of the cancer cells [47]. Different studies have shown that the expression of TGF-β-1 in CAFs activates different signalling pathways which drive several pro-tumoural processes in HCC [50,51]. Hepatocyte growth factor (HGF) is also highly expressed in CAFs and has been described to influence EMT and tumour survival-activating signalling pathways such as ERK and AKT in tumour cells [48]. Chemoresistance is a key problem in the development of new cancer therapies [52]. In HCC, CAFs were found to increase chemoresistance against sorafenib and cisplatin through the HGF-Met-ERK pathway [53,54]. Two other studies further confirmed that CAFs induce drug resistance against sorafenib, which in turn leads to an increase in liver cancer cell aggressiveness [55,56].

CAFs were found to facilitate the development of an immunosuppressive TME by inhibiting the infiltration of pro-inflammatory T-cells and by promoting the infiltration of immunosuppressive T-cells [57]. Moreover, CAFs are capable of inducing tumour-resident myeloid-derived suppressor cells [58], which have the capacity to inhibit the activity of cytotoxic T lymphocytes [59]. Angiogenesis is another essential step in tumour development. It is initiated by hypoxia and can be modulated by CAFs [60]. CAFs have been found to express VEGF [61] and angiopoietin 1 [62] and 2 [63], which activate receptors of endothelial cells in tumours to promote the formation of new blood vessels [60]. Lastly, Coulouarn et al. showed that the crosstalk between CAFs and HCC cells leads to an upregulation of pro-fibrogenic and pro-inflammatory cytokines (IL1β, IL6, and IL8), acute phase proteins (CP and SAA1), and growth factors (AREG and EREG), indicating that this crosstalk promotes a pro-inflammatory and pro-angiogenic TME, which might lead to enhanced migratory capacity of the HCC cells [61].

In conclusion, CAFs appear to be involved in every step of liver cancer development. At the moment, numerous promising compounds for the treatment of primary and secondary liver cancer fail during clinical trials [64]. This could be explained by the lack of CAFs in preclinical in vitro models. Consequently, the chemoresistance-promoting role of the CAFs is not considered when drugs are first tested, and thus they fail once tested in vivo [64].

## 4. Definition and Use of CAFs for In Vitro Liver Cancer Studies

For many years, CAFs were seen as a homogeneous group of myofibroblasts characterized by the expression of vimentin, fibroblast activating protein (FAP), and α-smooth muscle actin (α-SMA) [65]. The cellular origin of the CAFs cannot be determined based on these prototypic fibroblast markers. Since the definition of CAFs remains unclear, in this review, we will use the definition of Sahai et al., which states the following: “CAFs are defined as cells negative for epithelial, endothelial and leukocyte markers with an elongated morphology and no mutations” [66]. However, the definition by Sahai et al. does not allow distinguishing CAFs from common myofibroblasts including non-tumour-associated activated HSCs. In the liver, activated HSCs (aHSCs) are the main myofibroblast source upon injury and fibrogenesis [41,67]. These aHSCs express PDGF-bb, TGF-β, IL-6 and IL-1b. While liver CAFs do present with an increased expression of HGF, FGF, and VEGF, when compared to aHSCs [68], pan-CAF markers that were recently used for cluster definition on single cell RNA sequencing (sc-RNA seq) data include only genes that are also highly expressed by non-tumour- associated HSCs (Schwabe, Pan-CAF signature: Col1a1, Col1a2, Col3a1, C1s1, Acta2, C1ra, Serpinf1, Pdgfrβ, Col12a1) [9,11]. Because of the poor definition of CAFs, many researchers choose to use HSCs instead of CAFs when studying the role of fibroblasts in liver cancer (see Section 5 and Section 6).

In this review, we include studies using different fibroblast sources (Table 1) such as primary CAFs isolated from DEN-treated mice or patients by enzymatic digestion followed by a selection based on, e.g., PDGFR-α, α-SMA or FAP, and culture passaging, but also primary HSCs from mouse or human livers and HSC cell lines. The LX-2 cell line is the most used CAF surrogate for in vitro modelling of liver cancer [69,70]. Since the LX-2 cells are α-SMA, vimentin, and PDGFR-β positive and secrete factors such as pro-collagen, pro-MMP-2, and tissue inhibitor of metalloproteinases 2 (TIMP-2), they are considered to represent aHSCs [69,70]. They are abundantly available and are much more robust than primary cells especially compared to human cells where interpatient differences are highly likely. These features are general characteristics for immortal cell lines, and similar to the frequent use of LX-2 cells as CAF surrogate, cancer cell lines are mostly used as a surrogate for primary cancer cells in the co-culture studies that we found. The most frequently used cell lines are listed in Table 1 and have a doubling time of approximately 30 h. They are derived from a single patient and do not reflect the complexity of human cancer caused by inter- and intrapatient variability [71]. The culture conditions and related results from studies using these cell lines in co-culture are described in the next paragraphs.

In the past few years, new techniques such as sc-RNA seq have shown that heterogeneity also exists in CAFs. Pathway analysis suggests that different subtypes exert different, maybe even opposing functions [9,41,72]. These recent findings bring new challenges and opportunities to develop CAF or CAF subtype-targeting strategies to complement currently available anti-cancer therapies. To develop such strategies, in-depth analysis of these newly identified CAF subsets is essential, and this will require the adaptation of currently used in vitro culture systems or development of new models.

Cancer researchers use in vitro models as essential tools to screen for new therapies, and in recent decades, these in vitro models have evolved from simple 2D mono-cultures [84,85] to complex 3D co-culture systems [86,87,88,89,90,91,92]. Two-dimensional cultures allow for straightforward analysis of gene and protein expression changes or to perform functional tests such as migration, cell viability, angiogenesis, cell signalling, and ECM remodelling assays. To study the interaction between two cell types, for example between cancer cells and CAFs, transfer of conditioned medium (CM) or transwell studies are frequently used. These methods only allow the study of communication through secreted molecules and the culture of HSCs on plastic plates is known to strongly activate the cells and to substantially differ from in vivo activation [93,94]. Increasing efforts in the creation of spheroid/organoid cultures is ongoing and of particular interest as three aspects of solid tumours are recapitulated: 3D structure, presence of multiple cell types, and presence of ECM (either self-produced or by growth in Matrigel). The engineering of in vitro tumour models with stromal cells, such as CAF, TAM, or MSC, has been recently reported and clearly shows evidence of 3D superiority in terms of drug discovery [95,96]. While 3D multicellular (tumour) spheroid models are becoming an essential tool in cancer research, they are still associated with many challenges that need to be overcome before the use of these spheroids holds potential as a pre-clinical tool (these challenges are extensively reviewed by Han, S.J. [97]). In the two following sections, we present an overview of the different in vitro studies that have focussed on CAF changes in response to tumour cells for primary and secondary liver cancers.

## 5. Models for CAF-Tumour Interaction in Liver Cancer

For the in vitro study of CAFs in primary liver cancer, approximately 86% of the studies focused exclusively on the effect of CAFs on tumour growth, tumour survival, and tumour response to chemotherapy. Only eight studies so far (representing 14%) have investigated the effect of the tumour cells on the CAF population and of that two-thirds of the papers used the LX-2 cell line as CAF surrogate (Table 2).

The presence of cancer cell lines can promote the CAF phenotype in LX-2 as shown by the induction of α-SMA, MMP-9, VEGF, and FGF [55,61,73,78]. This is likely mediated by secreted factors as the majority of the papers used CM transfer, exosome transfer, or transwell assays (Table 2). The transition of HSCs to CAFs was described by different researchers using different HCC lines. The CAF transition is attributed to exosomal miR-21 [73], which induces a dose-dependent increase in α-SMA and FAP and enhances migration and proliferation of LX-2 cells and to BAFF/NFκB signalling in response to co-culture with Sorafenib resistant Huh7 cells [55]. Furthermore, HepG2 cancer cells can promote primary HSC migration by the release of PDGF-bb [74]. In a recent study, Zhang et al. investigated the influence of tumour cells on a specific CAF subtype they selected from a bio-informatics analysis on patient cholangiocarcinoma samples [72]. The authors found that the IL-6/IL-6R pair was enriched in the interactions between CD146+ vascular CAFs or vCAFs and malignant cells. Exosomes isolated from cholangiocarcinoma cultures induced IL-6 expression in the vCAFs, and the authors showed that exosomal miR-9-5p is responsible for this enriched IL-6 expression and release by the CAF compartment and in response to the enhanced IL-6, EZH2, and malignancy were induced in the tumour [72].

The enrichment of pro-fibrogenic and pro-inflammatory cytokines (IL-1β, IL-6 and IL-8), acute phase proteins (CP and SAA1), and growth factors (AREG and EREG) in CAFs by tumour cells was shown earlier in an LX-2 HepaRG transwell model [61]. In a 3D organoid model, created with primary CAFs and tumour cells from DEN-treated mice, CAFs formed net-like structures around the tumour cells [56]. In the same study, an increase in Gremlin-1 in the CAFs was observed after tumour organoid medium transfer, which suggests a role for pro-fibrogenic BMP signalling [56]. The most extensive in vitro analysis of CAFs in co-culture with liver hepatoma cell lines was performed recently by Myojin et al. [77]. They showed that co-culturing with hepatoma cells (HepG2, Hep3B, Huh7) increased GDF15 expression in the LX-2s, which was attenuated by knocking out ATG7 in the LX-2s. GDF15 was identified as 1 of 9 secreted proteins that showed the strongest change in expression by the co-culture. The authors validated the effects of tumour cells on LX-2 activation and GDF15 expression in a xenograft in vivo study and validated in 12 HCC patients that tumour-associated HSCs express higher levels of GDF15 than non-tumour HSCs [77].

These data indicate that the crosstalk between liver cancer cells and HSCs promotes a pro-fibrogenic, pro-inflammatory, and pro-angiogenic tumour microenvironment, which in turn might lead to an enhanced proliferative and migratory capacity of the cancer cells.

Finally, a popular and very interesting approach is the use of patient derived organoids (PDOs). PDOs are generated by dissociation of patient tumour tissues, after which the total cell suspension is mixed with ECM proteins in a gel for further culture. Current studies using PDOs have only looked at tumour cell growth and response to chemotherapeutic treatments. The effect of the CAFs in these PDOs is likely to be present, but simply neglected. However, the CAFs can be involved in the observed intratumor and interpatient heterogeneity as the CAF population too is heterogeneous in and between patients [98,99,100,101].

The limited number of manuscripts focussing on signalling from tumour to CAFs in 3D HCC cultures marks one of the important challenges associated with complex culture systems: the use of specific read-outs for the individual cell types that are incorporated in such 3D systems. Therefore, the focus is on easy-to-measure parameters such as spheroid size, total cell proliferation or apoptosis in the whole system. This finding opens opportunities for future developments in this field using for example cell type specific reporters or cell type specific markers that can be identified from the multitude of recently published sc-RNA seq studies.

## 6. In Vitro Models Studying the Role of HSCs in Liver Metastases

In liver metastases, HSCs are the most important cells involved in the promotion of tumour progression due to their recruitment and activation into CAFs in the TME [11]. One of the key processes during CRLM is the modulation of ECM and CAFs are the major source of collagen deposition in the TME [102]. In addition, they are involved in the degradation of the ECM through the secretion of MMPs, such as MMP-1 [103], MMP-2, and MMP-9 [104]. Besides ECM modulation, the activation of HSCs into CAFs in the TME suggests a role for several pathways involved in pro-tumoural processes. Several studies have focused on colorectal cancer cell-induced HSC activation and how the tumour–stroma interactions can modulate the metastatic capacity. Most of these studies used 2D in vitro culture models. Strikingly, 50% of the CRLM studies used primary HSCs as CAF, while in the primary liver cancer studies 75% of the papers used the LX-2 cell line (Table 2 and Table 3).

Several molecules and pathways are implicated in the tumour–stroma crosstalk in CRLM, and it seems that, independently from the CRC cell line used, TGF-β is a key factor. This was shown using organoid cultures, as well as tumour CM transfer from HCT-116 or HT-29 cells, and was found to affect LX-2 cells [76,82]. Tumoural CXCR-4 was found to promote the differentiation of HSCs into CAFs through the induction of SDF-1 expression in HSCs, resulting in TGF-β secretion by CRC cells, which further induced the HSC differentiation into CAFs and promoted liver metastases [82]. The CXCR-4 pathway is not only linked to TGF-β signalling, but also favoured T-cell hypo-responsiveness and thus HSCs also play an immunosuppressant role in the hepatic microenvironment and promote CRLM [105]. Besides TGF-β, tumoural PDGF-C could promote LX-2 activation as well, partly via PAK-2 signalling, which initiated a pro-tumour effect from the HSCs on the tumour cells [75].

ECM remodelling is essential in tumour development and metastatic processes, and it was recently shown that while CAF-secreted hyaluronan promotes metastatic tumour size, collagen 1 creates a physical barrier hampering tumour growth [11]. Like in fibrosis, HSCs and HSC-derived CAFs are an important source for ECM deposition [11], and this ECM–HSC interaction was shown to be promoted by TGF-β in LX-2-HCT116 organoids [76]. Further stiffening of the organoids was observed and resulted in increased α-SMA and FAP expression, suggesting HSC-to-CAF differentiation and affecting in turn the tumour growth and differentiation status [76]. The role of environmental stiffness was also shown in a 3D study using HSCs from patient CRLM tissue cultured in polyacrylamide gels with a precise stiffness [106]. Increased stiffness promoted HSC activation by activating a RHOA-AKT-p300 mechanosignalling cascade and promoted transcription of more than 20 tumour-promoting factors. While the authors evaluated the effect of HSC CM on tumour cell growth, they did not show how the tumour is involved in regulation of environmental stiffness and activation of the RHOA-AKT-p300 pathway [106]. RhoA is known to be involved in cell migration and recruitment of LSECs and HSCs, which is a crucial event for the establishment and growth of experimental CRC tumours [107].

Mueller et al. showed that CAF migration is promoted by the presence of tumour cells, although the factors that mediate this migration remain elusive [83]. Finally, the Arteta group showed that CT-26 CM promoted LSEC and HSC migration and that soluble ICAM-1 pre-treatment of tumour cells enhanced this effect [79,80]. They later showed that tumoural COX-2 was involved in the pro-migratory effects of soluble ICAM-1 [80].

The lack of studies exclusively focusing on CAFs in CRLM shows a poor knowledge of the interactions between CAFs and tumour cells. The assumption that the response of CAFs in CRLM is the same as in HCC is incorrect and should be rapidly discarded if we want to uncover the role of CAFs during liver metastasis. The use of 3D models with new techniques such as sc-RNA-seq could be the much-needed tool to increase our knowledge of CAFs in CRLM.

## 7. Conclusions and Future Steps in CAF-Tumour Interaction Studies

**Figure 2:**
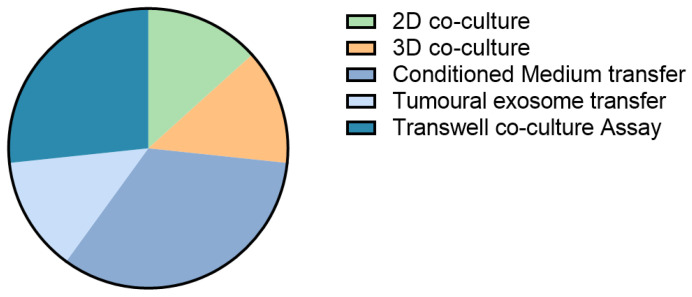
Culture setups to study the effects of tumour cells on CAFs in liver cancer.

To obtain mechanistic and biological insights on tumour–TME interactions in cancer progression, metastasis, and drug resistance, scientists have aimed to develop biologically relevant 3D in vitro models. Despite the fact that different cellular sources are being used for CAF-tumour in vitro studies, some common pathways are being described for CAFs in primary and secondary cancers such as the involvement of pro-fibrogenic growth factor TGF-β [56,76,78,82] and inflammatory cytokine IL-6 [72,83]. These are of course very common signalling cascades, and therefore therapeutic modulation could lead to severe side effects. Of the studies discussed, only 27% look at the effects of direct cell co-culture; the other 73% focus on the effects of cancer-secreted signals on the HSCs/CAFs (Figure 2). These secreted signals probably only reflect part of the actual in vivo communication between the investigated cell types, where direct cell–cell contacts exist to complement secreted signalling. Moreover, in only a minority of the studies, multiple cancer cell lines were used. The implementation of different cell lines would include a wider range of mutational, ethnic and sex backgrounds, which affects tumour growth and response to chemotherapy [71,81] and might also differentially influence CAF behaviour.

Although the role of CAFs in tumour aggressiveness and invasiveness is well understood, the fraction of co-culture studies that describe effects of tumour cells on CAFs is approximately 14%. Given the increased knowledge regarding the TME, including the existence of different CAF populations, the inclusion of primary CAFs or even CAF subtypes in controlled 3D in vitro cultures might further aid in investigating the bidirectional communication between tumour cells and different CAF subpopulations. Finally, inclusion of other cell types such as immune cells and endothelial cells will improve a further mimicking of solid tumours and behaviour of the cell types that compose the tumour and TME. The addition of more cell types will require optimization of cell isolation, culture, and analysis procedures. This could partly be achieved by the PDO technique, where a whole tumour is digested and reconstructed in vitro. The disadvantage of PDOs is that they include patient heterogeneity, and while it is highly attractive for personalized medicine approaches, it also brings up more challenges concerning sample size to ensure enough power for mechanistic studies.

The development of CAF in vitro models improved during the last few years with a shift from 2D cultures with transwell inserts or CM transfers to more complex 3D multicellular systems. Despite these advances, our current lack of understanding of CAF identity and behaviour and the relative simplicity of the culture settings hampers the progress needed to develop CAF-targeting strategies.

## Figures and Tables

**Table 1:** Sources for CAFs and cancer cells used for in vitro modelling of primary and secondary liver cancer.

Variation	Cell Name	Cellosaurus [67]	Species	Origin	References
CAFs	Primary CAF	NA	Mouse	DEN model	[56]
	Primary CAF	NA	Human	CCA, HCC	[56,72]
	LX-2	CVCL_5792	Human	Hepatic stellate cells	[55,61,73,74,75,76,77,78]
	Primary HSCs	NA	Mouse	Liver digest	[79,80]
	3T3-NIH	CVCL_KS54	Mouse	Fibroblast	[80]
Liver cancer cells [71]	HepaRG	CVCL_9720	Human	HCC	[61]
	Huh-7	CVCL_0336	Human	HCC	[55,77]
	HepG2	CVCL_0027	Human	Hepatoblastoma	[74,77]
	LM3	CVCL_D269	Mouse	Malignant neoplasms of the mouse mammary gland	[73]
	MHCC97-H	CVCL_4972	Human	HCC	[73]
	HEpB3	CVCL_0326	Paediatric Human	HCC	[77,78]
	HuCCT1	CVCL_0324	Human	cholangiocarcinoma	[72]
	FRH0201	Not mentioned	Human	cholangiocarcinoma	[72]
	RBE	CVCL_4896	Human	cholangiocarcinoma	[72]
	QBC939	CVCL_6942	Human	cholangiocarcinoma	[72]
CRC cell lines [81]	HCT-116	CVCL_0291	Human	Colon carcinoma	[76,82]
	LS174T	CVCL_1384	Human	Colon adenocarcinoma	[75]
	HT-29	CVCL_0320	Human	Colon carcinoma	[82,83]
	CT-26	CVCL_7254	Mouse	Colon carcinoma	[79,80]

**Table 2:** Overview of studies on CAF-tumour interaction for primary liver cancer.

Manuscript	Culture Model	Culture Conditions	CAF Source	Tumour Source	Main Result	Ref.
Zhou Y, 2018	Tumour cell exosomes transfer to 2D	CM was collected from HCC cells in 10 CM plates with DMEM 10% FBS after 48 h. Exosomes were isolated through untracentrifugation.	LX-2	LM3, MHCC97-H	Tumour cells facilitate the transition of HSCs into CAFs (increased α-SMA, FAP, FSP1, VEGF-α, MMP2, MMP9, bFGF and TGF-β) via miR-21 and AKT.	[73]
Zhang M, 2020	Tumour cell exosomes transfer to 2D	Exosomes were derived from the supernatant of ICC cells collected from 48h serum-free cultures by ultracentrifugation.	Passaged primary CD146+ cells	HuCCT1, FRH0201, RBE, QBC939	Tumour-cell-derived exosomal miR-9-5p induces IL-6 expression in vascular CAFs, which enhances ICC malignancy.	[72]
	Tumour sphere-CAF transwell	CAFs were cultured for 24 h in the upper insert of a transwell in α-MEM 10% FBS, moved to fresh wells and supplemented with tumour sphere culture medium. A total of 2000 tumour cells were seeded in each chamber of a 6-well plate in DMEM 0% FBS.		HuCCT1	Tumour-cell-derived exosomal miR-9-5p induces IL-6 expression in vascular CAFs, which increases tumour sphere formation.	
Coulouarn C, 2012	Tumour-CAF transwell	LX-2 and HepaRG cells were co-cultured in serum- and DMSO-free William’s E medium in 6-well plates with 1 µm pore size transwell inserts.	LX-2	HepaRG	Tumour cells induce the enrichment of pro-fibrogenic and pro-inflammatory cytokines, acute phase proteins, and growth factors in CAFs.	[61]
Lin N, 2015	Tumour-CAF transwell	HepG2 CM was added to the lower chamber and LX2 suspension (cultured in serum free DMEM) was added to the lower chamber. LX2 were incubated with the tumour cell CM for 24 h.	LX-2	HepG2	PDGF-bb release by HepG2 induces LX-2 migration	[74]
Gao L, 2021	Tumour CM transfer	(Sorafenib resistant) Huh7 cells were cultured in DMEM 10% FBS for 48 h after which CM was collected which was added to LX2 cells cultured in DMEM at a 1:1 ratio for at least 48 h.	LX-2	Huh7, Huh7-SR	Sorafenib-resistant tumour cells facilitate the transition of HSCs into CAFs (increased α-SMA and FAP expression) through the induction of BAFF/NFκB signalling in CAFs.	[55]
Wang C, 2021	Direct co-culture in 2D	Sulfatase 2 overexpressing Hep3B cells were co-cultured with LX2 cells in DMEM 10% FBS for 72 h.	LX-2	Hep3B	Sulfatase 2-overexpressing tumour cells promote HSC to CAF differentation (increased ACTA2, FAP, and POSTN) via TGF-β/SMAD3 signalling.	[78]
Myojin Y, 2021	Direct co-culture in 2D	LX2 cells were co-cultured with the same number of hepatoma cells for 48 h.	LX-2	HepG2, Hep3B, Huh7	Co-culturing HSCs with tumour cells induces GDF15 expression in HSCs.	[77]
Liu J, 2020	Direct co-culture in 3D	Tumour organoids were dissociated and co-cultured with CAFs (grown in 2D flasks) by sorting the cells in well plates containing mouse organoid basic medium (0% serum) and 1% Matrigel using FACS.	α-SMA+ FAP+ primary cells from DEN mice, HCC, CCA patients	Primary cells from DEN mice, HCC, CCA patients	Tumour medium transfer increases gremlin-1 expression in CAFs with a suggested role for BMP signalling.	[56]

**Table 3:** Overview of studies on CAF-tumour interaction for CRLM.

Manuscript	Culture Model	Culture Conditions	CAF Source	Tumour Source	Main Result	Ref.
Bandapalli OR, 2012	Tumour CM transfer	Supernatans from wild type or PDGF-C silenced LS174T cells (cultured in RPMI 10% FCS) was transferred to LX-2 cells (cultured in DMEM 1% FCS).	LX-2	LS174T	Tumour-derived PDGF-C promotes LX-2 activation through PAK-2 signaling.	[75]
Mueller L, 2010	Tumour CM transfer	CAFs were seeded in the upper chamber and HT-29 cells in the lower chamber of a Boyden chamber in DMEM 10% FBS.	Primary human CAFs	HT-29	CAFs express IL-6 and MCP-1 induced by tumour TNF-alpha.	[83]
Herrero A, 2021	Tumour CM transfer	3T3 cells were cultured in DMEM/F-12 10% FBS and HSCs and CT-26 in RPMI-1640 with 0% and 10% FBS, respectively. CM was collected from these cells after 24 h of culture in RPMI-1640 without FBS.	Primary mouse HSCs and 3T3	CT-26	Tumour cells promote the migratory capacity of HSCs through ICAM-1/COX-2.	[80]
Benedicto A, 2018	Tumour CM transfer	HSCs were cultured in serum-free DMEM and treated with CM of CT26 cells, which were cultured in RPMI-1640 1% FCS.	Primary mouse HSCs	CT-26	Tumour cells induce CXCR4 expression in HSCs which reduces the cytotoxic capacity of T cells.	[79]
Tan Hao-Xiang, 2020	Tumour-CAF transwell	HCT-116 or HT-29 cells were seeded onto a transwell membrane and LX-2 cells were grown in the lower chambers. Cells were incubated in RPMI-1640 2% FBS.	LX-2	HCT-116, HT-29	Tumours cells induce SDF-1 expression in HSCs and tumour cell-derived CXCR4 and TGF-β mediate the differentiation of HSCs into CAFs.	[82]
Dominijanni A, 2020	Direct co-culture in 3D	LX-2 and HCT-116 cells were cultured in DMEM 10% FBS followed by a co-culture in organoids.	LX-2	HCT-116	Activated HSCs (by TGF-β presence) modulate the stiffness of ECM and reduce the chemotherapy response.	[76]
